# The Cannabinoid Receptor 2 Protects Against Alcoholic Liver Disease Via a Macrophage Autophagy-Dependent Pathway

**DOI:** 10.1038/srep28806

**Published:** 2016-06-27

**Authors:** Timothé Denaës, Jasper Lodder, Marie-Noële Chobert, Isaac Ruiz, Jean-Michel Pawlotsky, Sophie Lotersztajn, Fatima Teixeira-Clerc

**Affiliations:** 1 INSERM U955, Institut Mondor de Recherche Biomédicale, Créteil, F-94000 France; 2 Université Paris-Est, Faculté de Médecine, UMR-S955, Créteil, F-94000 France; 3 INSERM U1149, Center for Research on Inflammation, Paris, F-75018, France; 4 Université Paris Diderot, Sorbonne Paris Cité, Laboratoire d’Excellence Inflammex, Faculté de Médecine, Site Xavier Bichat, Paris, F-75018, France

## Abstract

Kupffer cells, the resident macrophages of the liver, play a major role in the pathogenesis of alcoholic liver disease. We have previously demonstrated that CB2 receptor protects against alcoholic liver disease by inhibiting alcohol-induced inflammation and steatosis via the regulation of Kupffer cell activation. Here, we explored the mechanism underlying these effects and hypothesized that the anti-inflammatory properties of CB2 receptor in Kupffer cells rely on activation of autophagy. For this purpose, mice invalidated for CB2 receptor (CB2^Mye−/−^ mice) or for the autophagy gene ATG5 (ATG5^Mye−/−^ mice) in the myeloid lineage, and their littermate wild-type mice were subjected to chronic-plus-binge ethanol feeding. CB2^Mye−/−^ mice showed exacerbated alcohol-induced pro-inflammatory gene expression and steatosis. Studies in cultured macrophages demonstrated that CB2 receptor activation by JWH-133 stimulated autophagy via a heme oxygenase-1 dependent pathway. Moreover, JWH-133 reduced the induction of inflammatory genes by lipopolysaccharide in wild-type macrophages, but not in ATG5-deficient cells. The CB2 agonist also protected from alcohol-induced liver inflammation and steatosis in wild-type mice, but not in ATG5^Mye−/−^ mice demonstrating that macrophage autophagy mediates the anti-inflammatory and anti-steatogenic effects of CB2 receptor. Altogether these results demonstrate that CB2 receptor activation in macrophages protects from alcohol-induced steatosis by inhibiting hepatic inflammation through an autophagy-dependent pathway.

Alcoholic liver disease (ALD), a major cause of morbidity and mortality worldwide, includes a broad spectrum of disorders, ranging from the relatively benign fatty liver to more severe forms of liver injury, including alcoholic hepatitis, and end-stage liver diseases such as cirrhosis and hepatocellular carcinoma^1^. Fatty liver or steatosis, characterized by the accumulation of triglycerides in hepatocytes, is the most common and earliest response of the liver to chronic alcohol consumption. The mechanisms leading to fat accumulation in response to alcohol exposure have been extensively studied in animal models and include dysregulation of pathways associated with fatty acid synthesis, uptake, and oxidation, and triglyceride synthesis and export^1^. Recent findings have shown that not only hepatocytes, but also non-parenchymal cells may contribute to fat accumulation in alcoholic steatosis. In particular, activation of Kupffer cells, the resident macrophages of the liver, by gut-derived endotoxin leads to the acquisition of a pro-inflammatory phenotype^2^. The resulting release of cytokines directly contribute to the development of steatosis. In keeping with these data, depletion of Kupffer cells blunts the development of fatty liver^3^ and TNF-R1-knockout mice are protected from alcohol-induced steatosis^4^. Moreover, co-cultures of activated Kupffer cells with hepatocytes promote lipid accumulation into parenchymal cells both in the context of ALD^2^ and non-alcoholic fatty liver disease (NAFLD)^5^,^6^,^7^,^8^.

Cannabinoid CB2 receptors are G protein-coupled receptors predominantly expressed by immune cells, including macrophages, and bind lipophilic ligands, in particular the endocannabinoid 2-arachydonoylglycerol^9^. CB2 receptors predominantly display protective properties during liver injury. These effects largely rely on anti-inflammatory and anti-fibrogenic signals generated by CB2 receptors expressed in hepatic immune cells and/or hepatic myofibroblasts, with paracrine effects on hepatocytes, which do not express CB2. In particular, CB2 receptor limits liver fibrosis progression^10^ and inhibits the inflammatory process associated with acute ischemia/reperfusion^11^ and concanavalin A-induced hepatitis^12^. Moreover, CB2 receptor exerts beneficial effects on alcohol-induced inflammation and steatosis by inhibiting Kupffer cell activation^2^. Heme oxygenase-1 (HO-1) is the rate-limiting enzyme in the catabolism of heme into biliverdin, free iron, and carbon monoxide. HO-1 is a stress-inducible protein highly expressed in Kupffer cells and that displays potent protective effects in the liver against alcohol-induced liver injury^13^. We have previously shown that HO-1 mediates the anti-inflammatory properties of CB2 receptor^2^. However, the mechanism underlying this protective effect in Kupffer cells is not yet understood.

Macroautophagy (hereafter referred to as autophagy) is a pathway by which cytoplasmic material, including soluble macromolecules and organelles, is delivered to lysosomes for degradation^14^. This process involves the coordinated action of autophagy-related genes (ATG) that results in the formation of a double-membrane vesicle called an autophagosome that fuses with a lysosome to form an autolysosome in which the sequestered material is degraded by lysosomal enzymes. Autophagy has long been recognized as a stress response to nutrient deprivation to maintain energy homeostasis. However, autophagy plays a critical role in regulating a wide variety of pathophysiological processes. This pathway has been linked to diverse aspects of innate and adaptive immunity, including pathogen resistance, antigen presentation, tolerance and lymphocyte development, as well as the negative regulation of inflammation^15^,^16^. In particular, autophagy displays anti-inflammatory properties in macrophages, with resulting beneficial reduction of inflammation in diseases such as atherosclerosis, colitis, steatohepatitis and liver fibrosis^17^,^18^,^19^,^20^,^21^. In the current study, we further explored the mechanism underlying the protective effects of macrophage CB2 receptor against alcohol-induced liver injury. Using mice invalidated for CB2 or ATG5 in the myeloid lineage, we demonstrate that autophagy in macrophages is a key mediator of the anti-inflammatory and anti-steatogenic properties of CB2 receptor in a model of chronic-plus-binge alcohol exposure.

## Results

### Macrophages from CB2^Mye−/−^ mice display a pro-inflammatory phenotype in response to LPS

**Figure 1:**
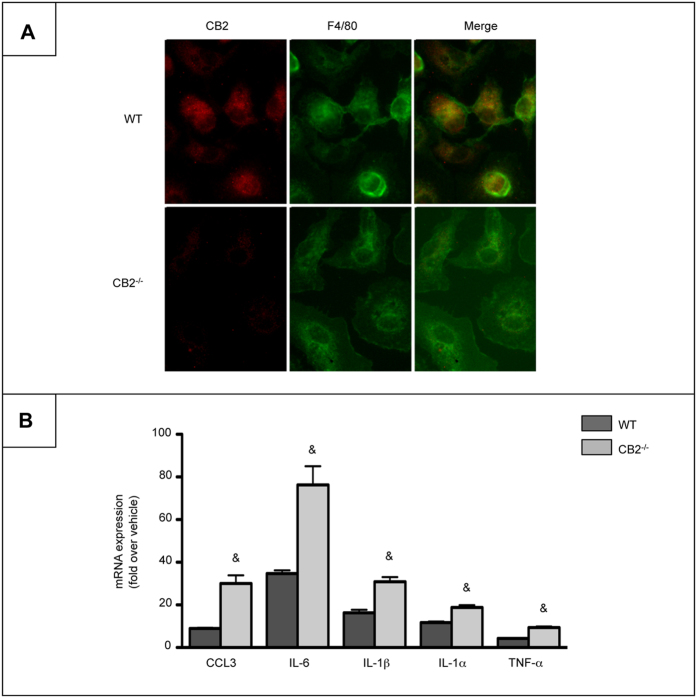
Macrophages from CB2^Mye**−/−**^ mice display enhanced pro-inflammatory properties. (**A**) Representative images of CB2 (red) and F4/80 (green) labeling in peritoneal macrophages isolated from WT and CB2^Mye**−/−**^ mice (original magnification x400). (**B**) mRNA expression of CCL3, IL-6, IL-1β, IL-1α and TNF-α in Kupffer cells isolated from WT and CB2^Mye**−/−**^ mice and exposed to 1 ng/ml of LPS for 6 hours. Data are mean ± SEM of 5–10 samples per condition. & *p* < 0.05 for WT vs CB2^Mye**−/−**^ mice.

We have previously demonstrated that CB2^**−/−**^ mice show exacerbated alcohol-induced hepatic inflammation and steatosis as compared to WT mice^2^. In order to directly assess the contribution of myeloid cells in these effects, we generated mice with a specific targeted deletion of CB2 receptor in myeloid cells (CB2^Mye−/−^) by crossing CB2^flox/flox^ mice with transgenic mice expressing the Cre recombinase under the control of the lysozyme M promoter (LysMCre^+/+^ mice), and monitored the efficiency of the deletion by immunocytochemistry, using thioglycollate elicited-peritoneal macrophages. As expected, CB2 receptor expression was immunodetected in WT but not in CB2^−/−^ cells (Fig. 1A). Moreover, Kupffer cells isolated from CB2^Mye**−/−**^ mice and exposed to LPS exhibited a pro-inflammatory phenotype, as shown by the enhanced expression of chemokine (C-C motif) ligand 3 (CCL3), interleukin-6 (IL-6), IL-1β, IL-1α and TNF-α in CB2^**−/−**^ cells as compared to macrophages from WT counterparts (Fig. 1B).

### CB2 receptor in myeloid cells protects against alcohol-induced inflammation and steatosis

**Figure 2:**
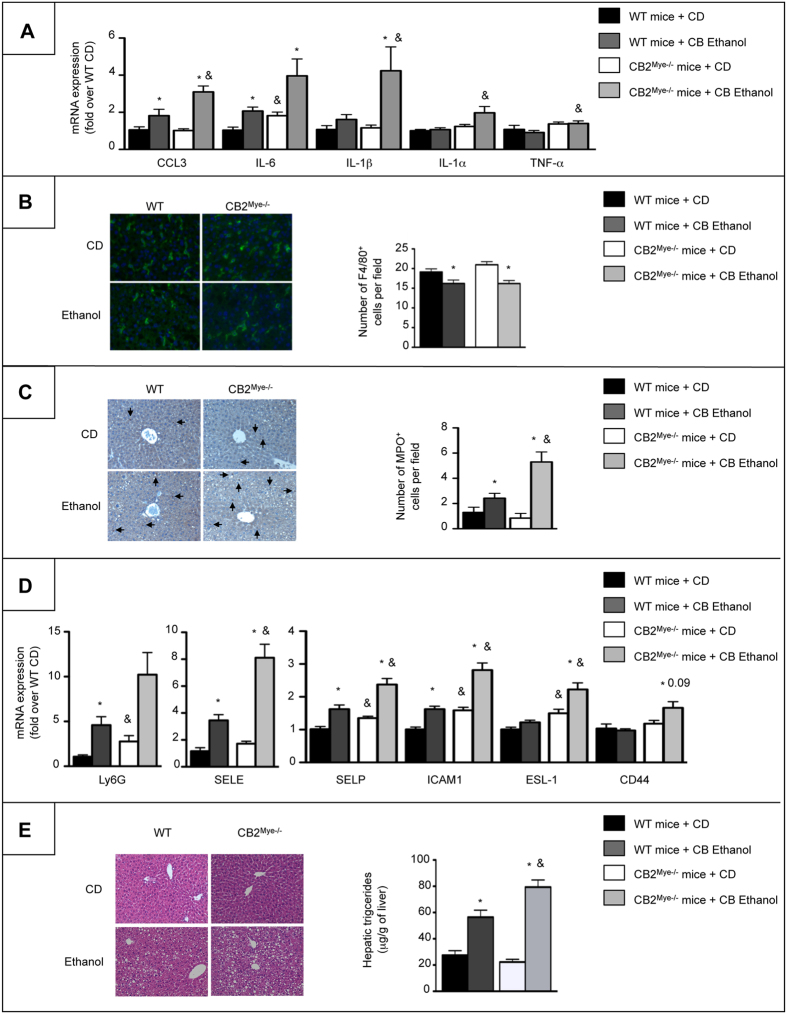
CB2^Mye**−/−**^ mice show enhanced liver inflammation and alcohol-induced steatosis. (**A**) Hepatic mRNA expression of CCL3, IL-6, IL-1β, IL-1α and TNF-α in control diet (CD)- and chronic-plus-binge ethanol-fed WT and CB2^Mye**−/−**^ mice. (**B**) Representative images (original magnification x400) and quantification of F4/80 staining in CD- and chronic-plus-binge ethanol-fed WT and CB2^Mye**−/−**^ mice. (**C**) Representative images (original magnification x200) and quantification of MPO staining in CD- and chronic-plus-binge ethanol-fed WT and CB2^Mye**−/−**^ mice. Arrows indicate positive cells. (**D**) Hepatic mRNA expression of Ly6G, SELE, SELP, ICAM1, ESL-1 and CD44 in CD- and chronic-plus-binge ethanol-fed WT and CB2^Mye**−/−**^ mice. (**E**) *Left*, representative hematoxylin-eosin staining of liver tissue sections from CD- and chronic-plus-binge ethanol-fed WT and CB2^Mye**−/−**^ mice (original magnification x200), and *right*, hepatic triglycerides content of CD- and chronic-plus-binge ethanol-fed WT and CB2^Mye**−/−**^ mice. Data are mean ± SEM. *p < 0.05 for CD vs ethanol and & p < 0.05 for WT vs CB2^Mye**−/−**^ mice.

We next investigated the contribution of macrophagic CB2 receptor to the beneficial effects of CB2 receptor on alcohol-induced inflammation and steatosis by subjecting CB2^Mye−/−^ mice and their WT littermates to chronic-plus-binge ethanol (CB Ethanol) feeding. As compared to WT counterparts, CB2^Mye−/−^ mice displayed exhacerbated pro-inflammatory response to alcohol feeding, as reflected by higher hepatic expression of pro-inflammatory markers, including CCL3, IL-6, IL-1β, IL-1α and TNF-α (Fig. 2A). We also evaluated the consequences of CB2 receptor invalidation in myeloid cells on inflammatory cells recruitment after alcohol exposure. The number of F4/80-positive cells was similar in ethanol-fed WT and CB2^Mye**−/−**^ mice (Fig. 2B). In contrast, CB2^Mye**−/−**^ mice showed a higher number of recruited neutrophils compared to WT mice in response to alcohol, as shown by myeloperoxydase (MPO) immunostaining (Fig. 2C). This increased neutrophil infiltration was associated with higher hepatic mRNA expression of the neutrophil marker Ly6G as well as a number of adhesion molecules such as E-selectin (SELE), P-selectin (SELP), intracellular adhesion molecule 1 (ICAM-1) and the selectin ligands E-selectin ligand 1 (ESL-1) and CD44 both at basal levels and following ethanol exposure (Fig. 2D). In addition, alcohol-fed CB2^Mye−/−^ mice showed enhanced lipid accumulation in hepatocytes at the histological examination and higher hepatic triglyceride content (Fig. 2E) but no difference in serum transaminase levels (Supplementary Fig. S1A) as compared to their WT counterparts. Taken together, these data indicate that CB2 receptor deficiency in myeloid cells enhances ethanol-induced activation of Kupffer cells, neutrophil recruitment and hepatic steatosis.

### CB2 receptor activation induces macrophage autophagy through an heme oxygenase-1 (HO-1) dependent pathway

**Figure 3:**
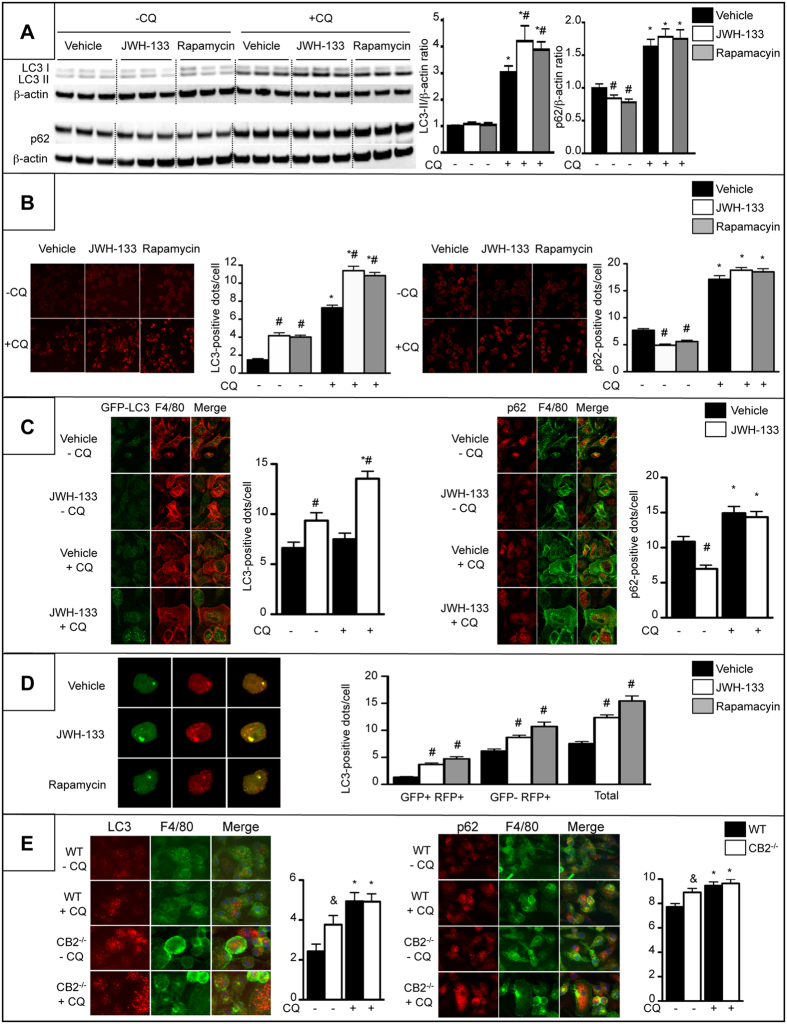
CB2 receptor activation induces autophagy in macrophages. Cells were exposed to 5 μM of JWH-133, 100 nM rapamycin or vehicle for 6 hours in the presence or absence of 10 μM of chloroquine (CQ). (**A**) Western blot analysis and quantification of LC3, SQSTM1/p62 and β-actin protein expression in RAW264.7 cells. (**B**) Representative images (original magnification x400) and quantification of the number of LC3-positive dots (*left*) and SQSTM1/p62-positive dots (*right*) in RAW264.7 cells. (**C**) Representative images (original magnification x400) and quantification of the number of GFP-LC3-positive dots in F4/80 (red)-positive peritoneal macrophages (*left*) and SQSTM1/p62 (red)-positive dots in F4/80 (green)-positive peritoneal macrophages (*right*) isolated from GFP-LC3 transgenic mice. (**D**) Representative images (original magnification x400) and quantification of LC3-positive dots in RAW264.7 cells transfected with the RFP-GFP-LC3 plasmid (autophagosomes = GFP^+^ RFP^+^ (yellow dots), autolysosomes = GFP^-^ RFP^+^ (red dots), Total = GFP^+^ RFP^+^ and GFP^-^ RFP^+^). (**E**) Representative images (original magnification x400) and quantification of the number of LC3 (red)-positive dots (*left*) and of SQSTM1/p62 (red)-positive dots (*right*) in F4/80 (green)-positive peritoneal macrophages isolated from WT and CB2^Mye**−/−**^ mice and exposed to 10 μM of chloroquine or its vehicle for 6 hours. Data are mean ± SEM. ^#^*p* < 0.05 for JWH-133 or rapamycin vs vehicle, *p < 0.05 for - CQ vs +CQ, & *p* < *0.05* for WT vs CB2^**−/−**^ cells.

Since autophagy is an anti-inflammatory process in macrophages^17^,^18^,^19^,^20^, we investigated whether autophagy may mediate the anti-inflammatory effects of CB2 receptor. We first characterized the impact of CB2 receptor on macrophage autophagy by quantification of Microtubule-associated protein light chain 3 (LC3)-II and Sequestosome-1 (SQSTM1)/p62 protein expression by western blot in RAW264.7 cells exposed to the lysosomal inhibitor chloroquine (CQ). In these experiments, the autophagy inducer, rapamycin, was used as a positive control. Treatment of macrophages with JWH-133 or rapamycin enhanced the accumulation of LC3-II in the presence of chloroquine (Fig. 3A). Moreover, both JWH-133 and rapamycin decreased SQSTM1/p62 levels in the absence of chloroquine while, as expected, SQSTM1/p62 accumulates in the presence of chloroquine (Fig. 3A). Autophagy flux was also monitored by quantification of the number of LC3- and SQSTM1/p62-positive dots per cell. Treatment of macrophages with JWH-133 or rapamycin enhanced the accumulation of LC3-positive dots, a marker of the number of autophagosomes, and co-treatment with chloroquine further increased the levels of LC3 puncta (Fig. 3B), indicating that CB2-induced autophagosomes were degraded in the lysosome. Moreover, CB2 receptor activation and rapamycin decreased the number of SQSTM1/p62 dots and as expected, autophagy inhibition with chloroquine favored SQSTM1/p62 accumulation both in vehicle and JWH-133- or rapamycin-exposed cells (Fig. 3B). These results were confirmed using thioglycollated-elicited peritoneal macrophages isolated from GFP-LC3 mice and exposed to JWH-133 and chloroquine (Fig. 3C). These data indicated that the autophagic flux is enhanced in JWH-133-treated macrophages, a finding further confirmed by the use of a pH-sensitive LC3-reporter construct containing both a RFP and a GFP fusion tag. In this assay, the autophagosomes appear yellow due to red and green fluorescence, while after fusion with a lysosome, the acidic pH quenches the GFP signal, and autolysosomes appear red. Using this approach, we found that JWH-133 and rapamycin cause an increase in both the number of autophagosomes (yellow dots, before fusion) and autolysosomes (red dots, after fusion with the lysosome) (Fig. 3D). In addition, CB2^**−/−**^ macrophages exhibited enhanced accumulation of both LC3 and SQSTM1/p62 puncta as compared to WT cells (Fig. 3E) suggesting a reduced autophagy flux that could be due to a lysosomal defects in these cells. Moreover, co-treatment of the cells with chloroquine further increased the levels of LC3 and SQSTM1/p62 puncta consistent with a partial inhibition of autophagy in cells invalidated for CB2 receptor (Fig. 3E). These data demonstrate that CB2 receptors induce autophagy in macrophages.

**Figure 4:**
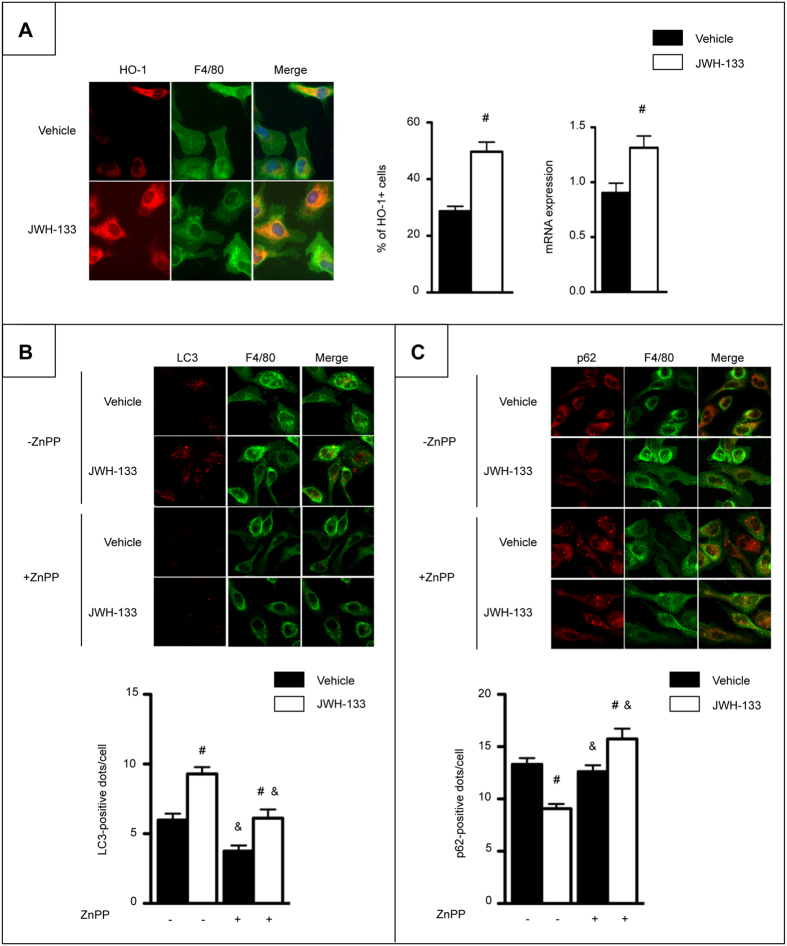
CB2 receptor activation induces autophagy in macrophages through an HO-1 dependent pathway. (**A**) *Left*, representative images (original magnification x400) and quantification of HO-1 (red) and F4/80 (green) double positive peritoneal macrophages isolated from WT mice and exposed to 5 μM of JWH-133 or its vehicle for 6 hours. *Right*, HO-1 mRNA expression in peritoneal macrophages isolated from WT mice and exposed to 5 μM of JWH-133 or its vehicle for 6 hours. (**B**) Representative images (original magnification x400) and quantification of the number of LC3 (red)-positive dots per F4/80 (green)-positive peritoneal macrophages exposed to 2 μM of ZnPP and 5 μM of JWH-133 or vehicle for 6 hours in the presence of 10 μM of chloroquine. (**C**) Representative images (original magnification x400) and quantification of the number of SQSTM1/p62 (red)-positive dots per F4/80 (green)-positive peritoneal macrophages exposed to 2 μM of ZnPP and 5 μM of JWH-133 or vehicle for 6 hours in the absence of chloroquine. Data are mean ± SEM. ^#^*p* < *0.05* for JWH-133 vs vehicle and & *p* < *0.05* for ZnPP vs vehicle.

Further experiments were designed to characterize the mechanism of CB2-mediated autophagy induction in macrophages. We have previously shown that the anti-inflammatory effects of CB2 receptor in macrophages are mediated *via* HO-1 induction^2^. We therefore investigated whether CB2 receptor activation of the autophagic pathway relies on HO-1 induction. JWH-133 induced HO-1 protein and mRNA expression (Fig. 4A). However, ZnPP, a specific competitive inhibitor of HO-1 activity prevented the formation of LC3 puncta (Fig. 4B) and SQSTM1/p62 degradation (Fig. 4C) in JWH-133-challenged cells, suggesting that CB2 receptor induces autophagy through an HO-1 dependent pathway.

### Activation of macrophage autophagy by CB2 receptors underlies the anti-inflammatory and anti-steatogenic properties of CB2 receptor

**Figure 5:**
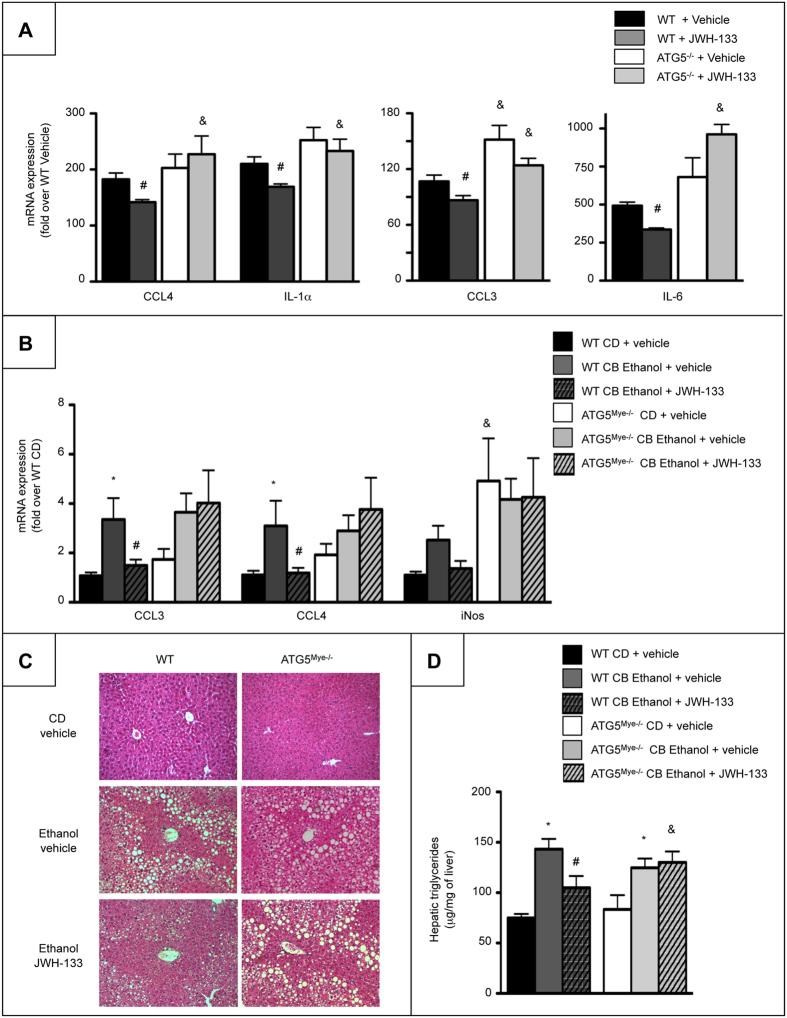
Macrophage autophagy mediates the anti-inflammatory and anti-steatogenic properties of CB2 receptor. (**A**) RT-PCR analysis of CCL4, IL-1α, CCL3 and IL-6 mRNA expression in peritoneal macrophages isolated from WT and ATG5^Mye**−/−**^ mice and exposed to 5 μM of JWH-133 or its vehicle for 24 hours and 10 ng/ml of LPS for the last 6 hours. (**B**) Hepatic mRNA expression of CCL3, CCL4 and iNOS in CD- and chronic-plus-binge ethanol-fed WT and ATG5^Mye**−/−**^ mice treated with 3 mg/kg of JWH-133 or its vehicle. (**C**) Representative hematoxylin-eosin staining (original magnification x200) and (**D**) hepatic triglyceride content of CD- and chronic-plus-binge ethanol-fed WT and ATG5^Mye**−/−**^ mice treated with 3 mg/kg of JWH-133 or its vehicle. Data are mean ± SEM. *p < *0.05* for CD vs chronic-plus-binge ethanol, & *p* < *0.05* for WT vs ATG5^Mye**−/−**^ and ^#^*p* < 0.05 for JWH-133 vs vehicle.

We next investigated whether autophagy may mediate the anti-inflammatory effects of CB2 receptor using ATG5-deficient macrophages exposed to JWH-133. ATG5 deficiency prevented the inhibitory effect of JWH-133 on the induction of the pro-inflammatory genes, CCL4, IL-1α, CCL3 and IL-6 in response to LPS (Fig. 5A). In order to further evaluate the *in vivo* contribution of the macrophage autophagic pathway in CB2 receptor-dependent effects, we examined the consequences of CB2 receptor activation on alcohol-induced steatosis in ATG5^Mye−/−^ mice. As shown in Fig. 5B to D, pro-inflammatory marker expression, steatosis and hepatic triglyceride content were reduced in ethanol-fed WT mice exposed to JWH-133 as compared with ethanol-fed WT mice, whereas these parameters were similarly increased in vehicle- and JWH-133-treated ATG5^Mye−/−^ mice. Interestingly, hepatic steatosis and inflammation were similar in ethanol-fed WT and ATG5^Mye**−/−**^ mice, consistent with the previously reported phenotype of ATG5^Mye**−/−**^ mice in a model of obesity-induced steatosis^20^. Moreover, alcohol-fed ATG5^Mye−/−^ mice showed no difference in serum transaminase levels as compared to their WT counterparts (Supplementary Fig. S1B). Altogether, these data demonstrate that the beneficial effects of CB2 receptor on alcohol-induced inflammation and steatosis are mediated through an autophagy-dependent pathway in Kupffer cells.

## Discussion

Our study uncovers macrophage autophagy as a major signaling pathway in the anti-inflammatory and hepatoprotective effects of CB2 receptor during alcoholic liver disease. Mechanistic studies also demonstrate the role of HO-1 in the induction of autophagy by CB2 receptor.

Accumulating evidences link inflammation to metabolic changes associated with fat deposition in alcoholic liver disease. In particular, Kupffer cells play a pivotal role in fat accumulation through their production of factors that control both metabolic and alcoholic steatosis by paracrine effects^3^,^4^,^5^,^6^,^7^,^8^. Therefore, identification of targets that limit Kupffer cell activation is now considered as an attractive hepatoprotective strategy in the context of alcoholic liver disease. Our previous data suggested that CB2 receptors may constitute a potential therapeutic target^2^. Here, we developed CB2^flox/flox^ mice and generated mice with a specific targeted deletion of CB2 receptor in myeloid cells. Exposure of these mice to chronic-plus-binge alcohol feeding leads to enhanced activation of Kupffer cells and results in exacerbated steatosis, demonstrating the direct contribution of CB2 expressed in macrophages to its anti-inflammatory and anti-steatogenic properties. These data also further argue for a determinant role of Kupffer cells in the control of hepatocyte steatosis.

Neutrophil accumulation in the liver is a common feature of alcoholic liver disease^1^ and is controlled by a multi-step adhesion cascade mediated by several adhesion molecules and their ligands expressed on endothelial cell and neutrophils, respectively^22^. In this study, we demonstrate that CB2 receptor in macrophages limits accumulation of neutrophils in response to alcohol feeding as well as the expression of the endothelium-expressed molecules, E- and P-selectin and ICAM1 and the neutrophil-expressed selectin ligands, ESL-1 and CD44. The mechanism by which CB2 receptor regulates neutrophil accumulation may involve CB2 receptor-mediated reduction of pro-inflammatory mediators, such as IL-1β, since IL-1β expression positively correlated with the hepatic expression of E-selectin^23^ and IL-1R-deficient mice show reduced neutrophilic infiltrate^24^.

Autophagy is a central regulator of lipid metabolism in hepatocytes and reduces alcoholic and metabolic steatosis by targeting lipid droplets for lipophagic degradation^25^,^26^. Interestingly, our results demonstrate that induction of macrophage autophagy by the CB2 receptor agonist reduces hepatic steatosis showing that the anti-steatogenic properties of autophagy are not restricted to hepatocyte. However, while induction of macrophage autophagy exerts beneficial effects on hepatic steatosis, ATG5^Mye**−/−**^ mice display a normal phenotype after chronic-plus-binge alcohol feeding. The mechanisms underlying this effect will need further investigations. It should be noted that althought activation of Kupffer cells plays a major role in fat accumulation, direct effects of alcohol on steatogenic pathways in hepatocytes are also involved and may explain the absence of phenotype of ATG5^Mye**−/−**^ mice. However, these data are consistent with a recent study demonstrating that loss of macrophage autophagy did not affect hepatic steatosis and inflammation in a model of obesity-induced steatosis^20^. Another major finding is that we unravel CB2 receptor as an inducer of autophagy in macrophages. Moreover, ATG5^Mye**−/−**^ mice are resistant to the anti-inflammatory and anti-steatogenic properties of JWH-133. These data uncover an additional novel function of CB2-induced autophagy in the regulation of inflammation in macrophages, in addition to its reported antitumoral properties in cancer cells^27^.

Mechanistically, we identify HO-1 as the mediator of autophagy induction by CB2 receptor. HO-1 is a stress-inducible protein with potent anti-inflammatory properties^28^. We have recently shown that up-regulation of HO-1 by CB2 receptor underlies its anti-inflammatory properties in Kupffer cells^2^. We show here that CB2-induced autophagy is blunted by the specific HO-1 inhibitor, ZnPP. These data provide a link between CB2 receptor activation and induction of macrophage autophagy and are consistent with a recent report showing that induction of autophagy in macrophages plays a major counteregulatory role in LPS-induced inflammation, via induction of HO-1^29^. Accumulating data suggest that HO-1 displays anti-inflammatory properties in the liver by dampening the pro-inflammatory response to LPS both in Kupffer cells and *in vivo* in the livers of ethanol-fed mice^13^. Accordingly, our data identify the HO-1/autophagy pathway as a signaling pathway by which CB2 prevents activation of Kupffer cells in response to chronic-plus-binge alcohol exposure.

In conclusion, the present study highlights macrophage autophagy as a major signaling pathway in the anti-inflammatory and anti-steatogenic properties of CB2 receptor in alcoholic liver disease. They also provide a rationale for the use of CB2 agonists as inducers of macrophage autophagy for limiting inflammation in the context of inflammatory diseases.

## Materials and Methods

### Materials

Ingredients for the Lieber-De Carli-modified diet were from MP Biomedicals or Sigma. The CB2 agonist JWH-133 was obtained from Axon Medchem, absolute ethanol from Carlo Erba Reactifs and Brewer Thioglycholate medium from BD Pharmingen. Lipopolysaccharide (LPS), Chloroquine (CQ), Rapamycin and Zinc Protoporphyrin (ZnPP) were from Sigma. The rabbit polyclonal anti-heme oxygenase-1 (HO-1) antibody was kindly provided by Dr Aïda Habib (INSERM U1149, Centre de Recherche sur l’Inflammation, Paris, France). The Red fluorescent protein-Green fluorescent protein-Microtubule-associated protein light chain 3 (RFP-GFP-LC3) plasmid was a kind gift from Dr Patrice Codogno (Inserm U1151, Institut Necker, Paris, France).

### Animals and experimental design

#### Animals

CB2^flox/flox^ mice were generated at the MCI/ICS (Mouse Clinical Institute/Institut Clinique de la Souris, Illkirch, France). The cnr2/cKO mutant mouse line was established using MCI proprietary 4.1 kb fragment (corresponding to the 5′ homology arm) and 4.8 kb fragment (corresponding to the 3′ homology arms). Fragments were amplified by PCR and subcloned in step1 plasmid to generate the final targeting construct. The linearized construct was electroporated in C57BL/6N mouse embryonic stem (ES) cells. After selection, targeted clones were identified by PCR using external primers and further confirmed by Southern blot with 3′ external probe. Two positive ES clones were injected into Balb/CN blastocysts, and male chimaeras derived gave germline transmission. We used the following primers to detect wild-type CB2 and CB2^flox^ alleles: TCCCAACAACAGGATACCCTGAGC and GGGGCCTCCAGAGATGATACTAAAGGA; CCAAGAGGCAAGGGTGACCTGA and CATACATTATACGAAGTTATCTGCAG. ATG5^flox/flox^ mice were kindly provided by Dr. Noboru Mizushima (Tokyo Medical and Dental University, Japan)^30^. Myeloid cell-specific CB2 (CB2^flox/flox^LysCre^+/−^, CB2^Mye**−/−**^) or ATG5 (ATG5^flox/flox^LysCre^+/−^, ATG5^Mye**−/−**^) deficient mice were generated by crossing lysozyme M-promoter Cre transgenic mice (Charles River, France) with CB2^flox/flox^ mice or ATG5^flox/flox^ mice, respectively. CB2^flox/flox^LysMCre^**−/−**^ and ATG5^flox/flox^LysMCre^**−/−**^ mice were used as littermate wild type (WT) controls. Green fluorescent protein (GFP)-LC3 transgenic mice were obtained from Riken BioResource Center (Tsukuba, Japan). Animals were housed in pathogen-free and temperature- and humidity-controlled animal facility and fed *ad-libitum.* All animal procedures were approved by the Committee for the Care and Use of Laboratory Animals of the Paris-Est Creteil University (ComEth, Authorization N°13-046 and 14-070). All animal research was carried out in accordance with the approved guidelines

#### Ethanol feeding protocol

Alcoholic liver injury was induced using the NIAAA model of chronic-plus-binge ethanol feeding^31^. Briefly, mice were fed the control Lieber-De Carli diet *ad libitum* for 7 days and then the ethanol Lieber-De Carli diet or pair-fed with the control diet for 10 days as previously described^2^. On day 18, ethanol and pair-fed mice were gavaged with a single dose of ethanol (5 g/kg body weight) or isocaloric dextrin-maltose and sacrificed 9 hours later. Mice were randomized into control diet (CD)- (n = 5 for WT and n = 6 for CB2^Mye**−/−**^) and ethanol-fed (n = 12 for WT and n = 16 for CB2^Mye**−/−**^) groups. The impact of the CB2 agonist, JWH-133, was evaluated in mice administered a daily intraperitoneal injection of JWH-133 (3 mg/kg) or its vehicle during the ethanol feeding period as previously described^2^. Mice were randomized into CD- (n = 5 for WT Vehicle and n = 6 for ATG5^Mye**−/−**^ Vehicle) and chronic-plus-binge ethanol-fed (n = 12 for WT Vehicle, n = 12 for WT JWH-133 and n = 16 for ATG5^Mye**−/−**^ Vehicle and n = 12 for ATG5^Mye**−/−**^ JWH-133) groups. Body weight and food intake were measured daily for all experiments and were similar between groups.

#### Hepatic triglyceride quantification

Hepatic triglycerides were extracted from 50 mg of liver homogenates by homogenization in 1 ml of acetone using TissueLyser (QIAGEN) and centrifugation for 10 min at 6000 rpm. Triglycerides were quantified with a triglyceride determination kit (Diasys) on liver samples.

#### Histological analysis

Liver specimens were fixed in 10% buffered formalin, embedded in paraffin and tissue sections (4 μm) were stained with hematoxylin-eosin.

#### Serum analysis

Aspartate aminotransferase (AST) and alanine aminotransferase (ALT) activities were measured on an automated analyzer in the Biochemistry Department (Henri Mondor Hospital).

### Cell culture

#### Kupffer cells

Kuppfer cells were isolated from WT and CB2^Mye**−/−**^ mice after perfusion with liberase (Roche) and differential centrifugation in Percoll (GE Healthcare) as previously described^2^. Adherent Kupffer cells were treated with 1 ng/ml LPS or its vehicle for 6 hours.

#### Peritoneal macrophages

Peritoneal macrophages were obtained from ATG5^Mye**−/−**^, CB2^Mye**−/−**^ and GFP-LC3 mice as previously described^19^. When indicated, cells were treated with 5 μM of JWH-133, 10 μM of chloroquine, 2 μM of ZnPP or vehicle for 6 hours.

#### RAW 264.7 macrophages

Cells were seeded in DMEM supplemented with 10% FBS. Cells were transfected with the RFP-GFP-LC3 plasmid using the Amaxa Cell line Nucleofector kit (Lonza) according to manufacturer’s instructions. 24 hours after transfection, cells were treated with 5 μM JWH-133 or 100 nM rapamycin or vehicle for 6 hours.

#### Western blot analysis

Western blot analysis was performed with the following antibodies : rabbit polyclonal anti-LC3b (1:500, Novus Biologicals), guinea pig polyclonal anti-p62 (1:1,000; ProGen), mouse monoclonal anti-β-actin (1:5,000; Sigma), followed by incubation with appropriate HRP-conjugated secondary antibodies (Jackson ImmunoResearch). The gels have been run under the same experimental conditions. Cells were treated with 5 μM JWH-133, 100 nM rapamycin, 10 μM of chloroquine or vehicle for 6 hours. Data are representative of 4 independent experiments with triplicate samples per experiment.

#### Immunohistochemistry

Immunohistochemical detection of F4/80 and myeloperoxidase (MPO) was performed as previously described^32^. The number of F4/80- or MPO-positive cells were quantified from 5–7 fields from 3–10 mice/group. F4/80 labeling was carried out in frozen liver sections fixed in 4% paraformaldehyde using a rat monoclonal anti-F4/80 antibody (1:20, Serotec). Labeling was achieved using a goat anti-rat IgG FITC (1:50, Serotec). No staining was observed when omitting the primary antibody. The number of F4/80-positive cells was quantified from 3–6 fields/animal and from 2–4 mice/group.

#### Immunocytochemistry

Cells were fixed in methanol, followed by incubation with a blocking buffer containing 1% BSA and 0.2% Triton X-100 and with a rabbit polyclonal anti-CB2 (1:200, Cayman), a rat anti-F4/80 antibody (1:20, Serotec), a mouse anti-LC3B antibody (1:200, Nano Tools), a guinea pig polyclonal anti-SQSTM1/p62 antibody (1:100, ProGen) or a rabbit anti-HO-1 antibody (1:300) and the appropriate secondary antibodies (goat anti-rabbit IgG Alexa 555 (1:1,000; Invitrogen), goat anti-rat IgG FITC (1:50, Serotec), goat anti-mouse IgG Alexa 555 (1:1,000; Invitrogen), goat anti-Guinea pig IgG Alexa 555 (1:1,000; Invitrogen)) as previously described^19^. Nuclear staining was performed using Prolong Gold antifade reagent with DAPI (Invitrogen). Fluorescence was imaged on a Zeiss LSM-510 multitracking laser scanning confocal microscope with a Helium/Neon laser at 543 nm and using AxioVision software (Carl Zeiss). No staining was observed when omitting the primary antibody. F4/80- and HO-1-positive cells from 10 fields/conditions were quantified. Results are expressed as percent of HO-1-positive cells per field. The number of SQSTM1/p62 or LC3-positive dots per F4/80-positive cell was quantified from 3–10 fields/condition.

#### RNA preparation and real time polymerase chain reaction

Total RNA was extracted using RNeasy Mini kit (QIAGEN). cDNA was synthesized using High-Capacity cDNA Reverse Transcription Kit (Life Technologies). Quantitative real time-PCR was performed using QuantiTect SYBRR Green PCR kit (Qiagen) on a Light Cycler 480 (Roche Diagnostics). Oligonucleotide primers for PCR were from MWG Biotech and amplified products were analyzed on a 2% agarose gel and sequenced. Sequences of primers for RT-PCR are given in Table 1.

#### Statistical analysis

Results are expressed as the mean ± standard error of the mean (SEM) and were analyzed by Mann-Whitney test using Prism 5.0 software (GraphPad). *p* < 0.05 was taken as the minimum level of significance.

## Additional Information

**How to cite this article**: Denaës, T. *et al*. The Cannabinoid Receptor 2 Protects Against Alcoholic Liver Disease Via a Macrophage Autophagy-Dependent Pathway. *Sci. Rep.* **6**, 28806; doi: 10.1038/srep28806 (2016).

## Supplementary Material

Supplementary Figure S1

## Figures and Tables

**Table 1:** List of mouse primer sequences used in quantitative PCR.

Gene	Sense	Antisense
18S	5′-AACTTTCGATGGTAGTCGCCGT-3′	5′-TCCTTGGATGTGGTAGCCGTTT-3′
CCL3	5′-TGAGAGTCTTGGAGGCAGCGA-3′	5′-TGTGGGTACTTGGCAGCAAACA-3′
CCL4	5′-AACAACATGAAGCTCTGCGT-3′	5′-AGAAACAGCAGGAAGTGGGA-3′
CD44	5′-TGAAACATGCAGGTATGGGT-3′	5′- GCTGAGGCATTGAAGCAATA-3′
ESL-1	5′-CAAGATGACGGCCATCATTTTCA-3′	5′-GTGCATCCTTTTCCCCAAGA-3′
F4/80	5′-CTTTGGCTATGGGCTTCCAGTC-3′	5′-GCAAGGAGGACAGAGTTTATCGTG-3′
ICAM1	5′-CAATTTCTCATGCCGCACAG-3’	5′-AGCTGGAAGATCGAAAGTCCG-3′
IL-1α	5′-ACGTCAAGCAACGGGAAGAT-3′	5′-AAGGTGCTGATCTGGGTTGG-3′
IL-1β	5′-CTCCACCTCAATGGACAGAA-3′	5′-GCCGTCTTTCATTACACAGG-3′
IL-6	5′-GAACAACGATGATGCACTTGC-3′	5′-TCCAGGTAGCTATGGTACTCC-3′
iNos	5′-AATCTTGGAGCGAGTTGTGG-3′	5′-CAGGAAGTAGGTGAGGGCTTG-3′
Ly6G	5′-TTGTATTGGGGTCCCACCTG-3′	5′-CCAGAGCAACGCAAAATCCA-3′
SELE	5′-AGCAGAGTTTCACGTTGCAGG-3′	5′-TGGCGCAGATAAGGCTTCA-3′
SELP	5′-GCCAGTTCATGTGCGATGAA-3′	5′-GGCGAAGATTCCTGGACACTT-3′
TNF-α	5′-AATGGCCTCCCTCTCATCAGTT-3′	5′-CCACTTGGTGGTTTGCTACGA-3′
