# Supplementary material for: The Cannabinoid Receptor 2 Protects Against Alcoholic Liver Disease Via a Macrophage Autophagy-Dependent Pathway
Source: Sci Rep. 2016 Jun 27;6:28806. doi: 10.1038/srep28806 (PMC4921859; doi:10.1038/srep28806)
Supplement: Supplementary Figure S1 [file srep28806-s1.pdf]

The Cannabinoid receptor 2 protects against alcoholic liver disease via a macrophage autophagy-dependent pathway

Timothé Denaës[1,2], Jasper Lodder[1,2], Marie-Noële Chobert[1,2], Isaac Ruiz[1,2], Jean-Michel Pawlotsky[1,2], Sophie Lotersztajn[1,2,3,4,*], Fatima Teixeira-Clerc[1,2,*]

[1] INSERM U955, Institut Mondor de Recherche Biomédicale, Créteil, F-94000 France

[2] Université Paris-Est, Faculté de Médecine, UMR-S955, Créteil, F-94000 France

[3] INSERM U1149, Center for Research on Inflammation, Paris, F-75018, France

[4] Université Paris Diderot, Sorbonne Paris Cité, Laboratoire d'Excellence Inflammex, Faculté de Médecine, Site Xavier Bichat, Paris, F-75018, France

* corresponding authors

Correspondence:

Dr. Fatima Teixeira-Clerc

INSERM U955, Institut Mondor de Recherche Biomédicale

Hôpital Henri Mondor, 94000 Créteil, France

Tel: +33 (0)1 49 81 35 37; Fax: +33 (0)1 48 98 48 44;

e-mail: fatima.clerc@inserm.fr

or

Dr Sophie Lotersztajn

INSERM U1149, Center for Research on Inflammation

Faculté de Médecine Xavier Bichat

16 rue Henri Huchard, 75018 Paris, France

Tel: +33 (0)1 57 27 74 35

e-mail: sophie.lotersztajn@inserm.fr

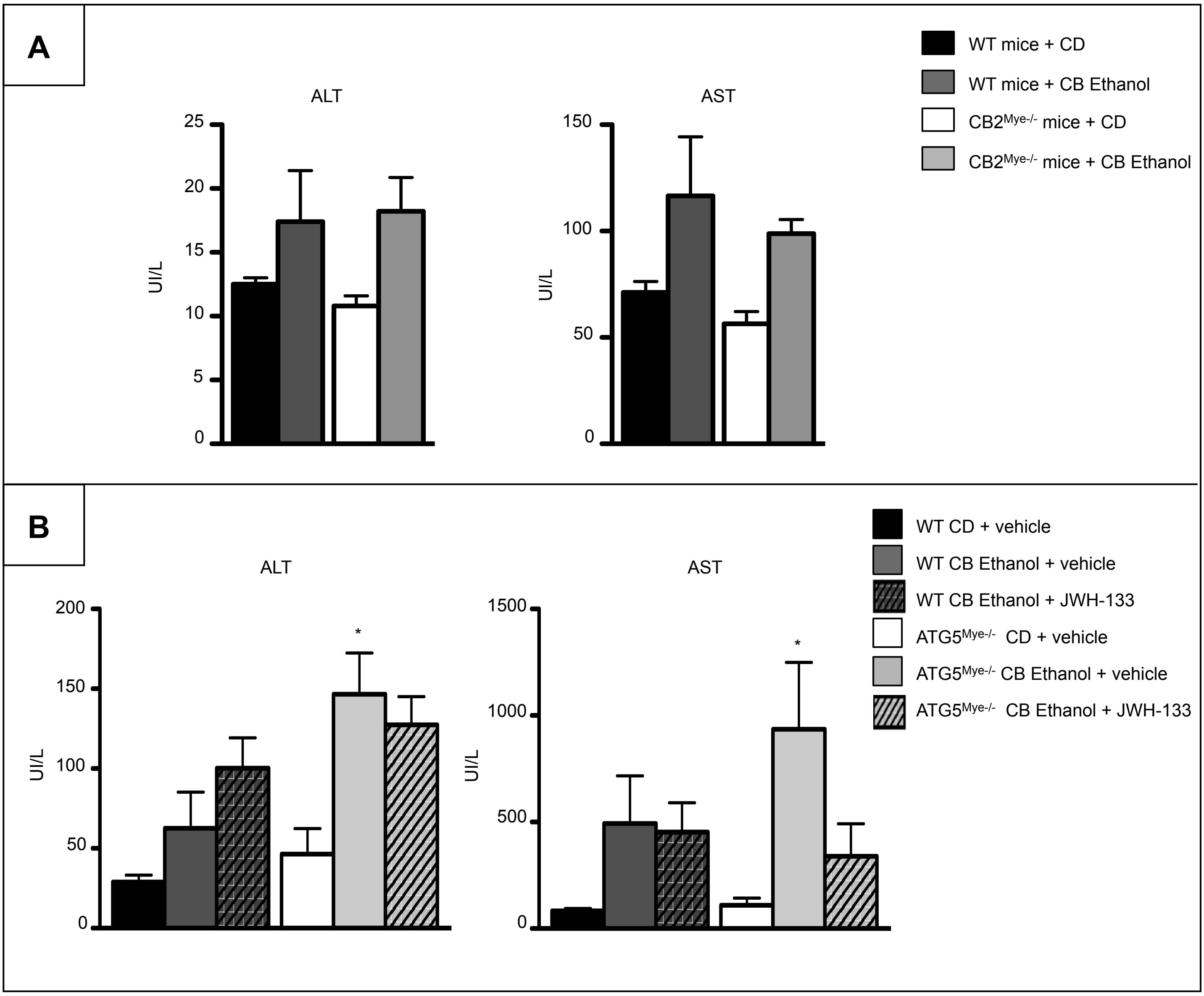

Supplementary Figure S1. Alanine aminotransferase (ALT) and aspartate aminotransferase (AST) levels (A) in CD- and chronic-plus-binge ethanol-fed WT and CB2$^{Mye-/-}$ mice and (B) in CD- and chronic-plus-binge ethanol-fed WT and ATG5$^{Mye-/-}$ mice treated with 3 mg/kg of JWH-133 or its vehicle. Data are mean ± SEM. * *$p<0.05$ for CD vs chronic-plus-binge ethanol.*
